# ‘Terminal anorexia’: a lived experience perspective

**DOI:** 10.1186/s40337-023-00823-x

**Published:** 2023-07-03

**Authors:** Alykhan Asaria

**Affiliations:** London, UK

**Keywords:** Ambivalence, Anorexia nervosa, Avoidance, Bully-friend, Cognitive, Diagnosis, Eating disorder, Hope, Identity, Label, Longstanding, Meaningful, Purposeful, Sufferer, Terminal, Terminal anorexia, Treatment, Treatment-refractory, Treatment-resistant

## Abstract

Having suffered from longstanding anorexia nervosa (AN) for more than a decade, and after meeting many patients who have also been labelled as ‘treatment-resistant’, ‘treatment-refractory’, or similar terms, I feel a strong responsibility to express my deep fears and sadness about the more harmful new label of ‘terminal anorexia’. This article is based on a reflective and private email that I emotionally wrote in Autumn 2022, soon after reading a thought-provoking paper (Guarda et al. in J Eat Disord 10:79, 2022) about the new term. When I wrote the email, I had not read the Gaudiani et al. (J Eat Disord 10:23, 2022) paper that proposed clinical characteristics for the new diagnosis. Hence, my email was not, and this article is not, a response to Gaudiani et al. (2022). Challenging the criteria that they proposed is beyond the scope of this article, which is just a lived experience reaction to the *concept* of ‘terminal anorexia’ (regardless of who created it and who tries to define it).

Before learning about ‘terminal anorexia’ in 2022, I assumed that ‘unconditional positive regard’ included mental health professionals’ unconditional hope for their patients’ ability to live meaningful lives, irrespective of how severe or chronic their patients’ conditions were. Therefore, I was very disheartened when the label ‘terminal anorexia’ was circulated by professionals. Research is not just read, seen, and heard about by the professionals who promote it. Vulnerable and conflicted eating disorder (ED) sufferers, and their families, can be victims of theoretical academic discourse that has real-world, life-or-death implications.

The purpose of my article is not to suppress the arbitrary new term, which is sadly already commonly used in clinical practice and amongst very young ED sufferers, despite it having no agreed definition. I intend to outline some of the reasons why I believe that the term (not its hypothesised criteria, which are beyond the scope of my article) is harming ED sufferers, so that these harms can be addressed before it is too late. I have grouped these reasons into six key themes that inevitably overlap and cannot be perfectly separated. They are: [1] Hope and identity destruction; [2] Avoidance and collusion; [3] Self-diagnosis and misdiagnosis; [4] Comparisons; [5] Dangerous precedents; [6] Current and future treatments.

## Hope and identity destruction

### Sufferers

The possibility that AN can be terminal, even if it is caveated with messages that this is rare, is extremely disheartening to sufferers who are already losing hope.^1^ Their pessimistic belief systems and cognitive biases (especially *labelling*, all-or-nothing thinking, overgeneralising, selective attention, and arbitrary inference), which are often strongly reinforced by the way that they have been treated/discussed/labelled by clinicians for years, mean that many will assume they have death sentences and can never get better.

I appreciate that some AN sufferers would feel relief if they were diagnosed with ‘terminal anorexia’. It can be a comforting and validating diagnosis. They may self-identify with the term, and finally feel understood by others. I have at times struggled not to relate to the term. In early 2022, I probably would have wanted to be diagnosed with ‘terminal anorexia’, and therefore given permission to die (or at least be relieved by the prospect of it). However, although I accept that I will never be ‘cured’ in the traditional sense, I now hope that I can have a purpose that makes life worth living, even if anorexia is always *in* my life—which is fundamentally different to it *being* my life (and death). I did not have this hope in early 2022 when my BMI was much lower. Notably, I did have capacity at the time—it is possible to have capacity and be confused/conflicted at the same time.

I have met AN sufferers in their 50s and 60s, with far worse experiences than mine and countless hospital admissions, who are living meaningful and purposeful lives. They are certainly not just ‘existing’ or waiting to die, though they may feel this way at times. Even the most ill and longstanding AN sufferers do not have to die or just ‘survive’ reluctantly. Opposing messages, which suggest that this is not possible, are readily accepted by individuals who are vulnerable and conflicted.

Unfortunately, it can take years for sufferers to realise that life—true living—is both possible and worthwhile. I have only just started this journey of discovery, and I expect that there will be many times when the AN bully-friend befriends me again, as there seems to be nothing else to fall back on. Until other sufferers can also start this journey, they need clinicians, and their loved ones if possible (who are just as much the victims of hope-crushing AN), to hold the hope for them.

Sufferers do not have to be controlled (or in this case killed) by their AN. This is because they have identities—often suppressed for many years—that go far beyond their EDs. They should not have to feel that defeating their ED requires defeating their whole selves. And they should always have the hope and knowledge—often hidden/silent—that they can and deserve to live (not just ‘exist’ or wait to die).

Labels such as ‘treatment-resistant’,^2^ ‘treatment-refractory’, and now ‘terminal anorexia’, can destroy hope and identity for those who receive them—even if these labels have not been formally ascribed or approved by treating professionals. Additionally, they can cause iatrogenic harm by inadvertently pressurising the labelled sufferer to fulfil the new ‘role’ (a ‘dying role’) that has been permanently assigned to them. A terminal diagnosis can implicitly instruct ED patients to wait for their certain deaths, rather than allow them to have the continued choice and hope of living.

Moreover, conflicting and inconsistent messages from different (or even the same) professionals—e.g., “You are more than your anorexia and can live a meaningful life,” versus, “You have no control over your life and anorexia,”—are extremely confusing for sufferers who are already ambivalent. It may undermine their trust in all professionals, thereby reinforcing the belief that only the AN can be relied upon.

Furthermore, the prolonged anxiety, uncertainty, and even unjustified guilt caused to a sufferer who learns about another patient’s ‘terminal anorexia’ diagnosis would be very distressing. Expecting someone they know—perhaps a fellow patient and/or a friend with shared experiences—to die from the same condition, yet not knowing for sure if or when this will happen, could be unbearable. It may even encourage the traumatised sufferer to cope by seeking/accepting the same fate as the patient diagnosed with ‘terminal anorexia’.

### Loved ones and carers

Hope is also crucial for the sufferer’s family, friends, and carers. They are also the victims of AN’s demoralising power. They may become so dejected by their loved one’s ‘terminal anorexia’ diagnosis, or just the suggestion/consideration of one, that they lose hope and *compassionately* give up on trying to fight to keep their loved one alive. They probably would have spent many years dedicated to this purpose.

For AN sufferers, having (or just perceiving that) those closest to them—probably their strongest ‘protective factors’ against death—are losing hope may in some cases be devastating. It may be ‘the final straw’, leaving them with no more reasons to stay alive—‘I might as well die now.’

## Avoidance and collusion

As stated earlier, receiving a ‘terminal anorexia’ diagnosis can provide temporary consolation to some AN sufferers. Fighting the ‘internal’^3^ AN ‘bully-friend’, and therefore fighting for life, is emotionally exhausting. Hence, being told that the fight will end soon can be a huge relief to sufferers who are conflicted or have already accepted death. The certainty of death can seem less unappealing than the uncertainty of life. Death can seem to be the only way out, just like a suicidal person with tunnel vision may see no other escape route.

However, temporary relief from distressing thoughts/emotions is not always helpful. As in anxiety disorders and OCD, safety behaviours can be used to avoid confronting distressing cognitions. Therefore, the sufferer cannot test the reality of those cognitions, and they cannot learn that there may be less catastrophic alternative outcomes. Unlike patients with anxiety-based disorders, patients diagnosed with ‘terminal anorexia’ would not get a second chance to test their negative assumptions after they have died.

Professionals who diagnose ‘terminal anorexia’ may inadvertently be agreeing with the sufferer’s AN ‘bully-friend’, which may tell them that because they cannot live without AN, they have no choice but to die from it. In this way, the internal AN ‘bully-friend’ may unintentionally be given an external ally, thereby feeding the very thing that starves the sufferer of hope and food.

## Self-diagnosis and misdiagnosis

Even if ‘terminal anorexia’ were only diagnosed in a tiny proportion of AN sufferers, what about the many other sufferers who would (and have already) self-diagnose (d) with the arbitrary condition, despite them having illnesses that can be treated or at least managed.

Moreover, what about the many sufferers who are misdiagnosed by well-meaning clinicians? Giving them a terminal diagnosis removes any hidden/silent hope that they may have. A sufferer who protests that there is no hope left may actually need the clinician to hold the hope for them, until they can carry it on their own. They may not feel deserving of the hope, or not know how to identify and express it. They may need clinicians to give them permission to live rather than permission to die.

Furthermore, when a ‘terminal anorexia’ diagnosis is being considered, who has the right to make the final decision? For example, is the opinion of a psychiatrist in a poorly resourced health service more important than that of a psychiatrist in a health service offering high-quality treatments? Is the opinion of a psychiatrist who has not worked with older AN sufferers more important than that of a psychiatrist who has witnessed remarkable improvements in patients who have lived with AN for decades? Is any psychiatrist’s opinion more important than that of a mother who has been fighting for years to keep her child alive?

## Comparisons

Counterintuitively, the ‘terminal anorexia’ diagnosis may become a ‘goal’ for sufferers with very low self-esteem, who may pin their self-worth on how ill/underweight they can make themselves compared to others. Young ED sufferers, especially those who are struggling to find their identities, are particularly vulnerable to this self-destructive paradox. To them, ‘achievement’ and ‘control’ may become defined by measurable (but meaningless) criteria, such as how much they can eat and weigh.^4^ Society encourages this tragic paradox by associating weight with perfection and control, and by only helping those who weigh below a certain BMI.

Patients who seek a ‘terminal anorexia’ diagnosis, but do not receive one, may feel that their illnesses are not being taken seriously, or that they are not as ill and deserving as patients who are given one. They may feel that they *need* to make themselves more ill, or even die, in order to be worthy of others’ care and validation.^5^

## Dangerous precedents

If AN can be defined as ‘terminal’, then surely any mental illness can be ‘terminal’? All mental illnesses can lead to premature deaths from suicide, self-harm, self-neglect, or associated physical health problems. A sufferer of severe depression is not diagnosed with ‘terminal depression’ if they may die soon from a cardiovascular condition resulting from inactivity, due to being trapped at home for many years. A sufferer of binge eating disorder (BED) is not diagnosed with ‘terminal BED’ if they may die soon from an obesity-related condition. Dying from the physical complications associated with a mental illness is not necessarily the same as dying from the mental illness itself (unless it is assumed that the whole person *is* the mental illness).

## Current and future treatments

So much time and research resources are being spent trying to create arbitrary criteria for a proposed condition that, in my opinion, cannot be defined. There is a huge opportunity cost, in terms of the wasted potential of research that could instead focus on treatments to help longstanding AN sufferers *live* (and not just ‘survive’, ‘exist’, or wait to die).

Palliative care is essentially ‘comfort/compassionate care’, which all AN sufferers deserve, regardless of their life expectancies. Research should focus on making sure that life-preserving treatments are based on just as compassionate principles. Patients should not have to be labelled as ‘terminal’ in order to be afforded the kindness, dignity, and respect that good palliative care intends to provide to individuals with terminal physical illnesses.

## Data Availability

There is no research data associated with this article, as it is based only on lived experiences.

## Abbreviations

AN: Anorexia nervosa
BMI: Body mass index
ED: Eating disorder

## Footnotes

1 While producing this article, I also realised that the ‘terminal anorexia’ debate is having a significant emotional impact on clinicians who work with AN patients. For example, some feel helpless and question what their roles are. Do they save patients who they genuinely believe can eventually live meaningful lives? Do they let their patients die because it seems that hope is gone? Do they get stuck fighting with colleagues in the muddy in-between grounds, waiting indefinitely for a consensus that ends the stalemate?

2 Apparent ‘treatment-resistance’ is often caused when patients are, or have historically been, treated in a way that encourages resistance. Regardless of the type of treatment provided, *all* professionals need to *consistently* apply it in a compassionate and empathic way that appreciates the patient’s true identity, rather than respond impatiently to the patient’s *illness*. Professionals must ask themselves whether the real problem is the delivery of treatment or the patient’s personal ‘resistance’ to it. I strongly believe that forced treatments should not be necessary when patients refuse to eat or be fed. There are more compassionate approaches. For example, a non-combative approach might involve the clinician just being with the patient who refuses to eat, showing genuine and unconditional compassion and empathy, actively *listening* to the patient if they choose to talk, and holding the hope for them until they can realise that their life is worth living and their body is worth nourishing (even if, initially, they only agree to this for the clinician who is willing to sit patiently and unconditionally with them). Even when an approach like this does not lead to the desired clinical outcome, it costs far less than forced and repeated medical interventions that have harmful outcomes. Unconditional patience costs far less than impatient reactions.

3 I have used the word ‘internal’ to convey and validate how ED sufferers often feel. However, it can sometimes be helpful for them to externalise and create distance from their EDs, in order to feel empowered by their more self-compassionate selves. In my view, there should be a balance between acknowledging how attached the sufferer may feel to their ED, especially if it genuinely helps them temporarily when they have nothing else to fall back on, and encouraging the sufferer to see their ED as something that should be ‘removed’ permanently because it is wholly evil.

4 In reality, severe EDs are usually driven by issues such as control, identity, and self-esteem. Food and weight typically only touch the surface of emotional conflicts that are much deeper. This highlights the importance of: [1] Prevention and early intervention, so that early eating difficulties do not progress deeper over time, through self-reinforcement and external reinforcement (including the attribution of unnecessary labels). [2] Specialist training for clinicians, so that they understand not just how, but also *why* to respond compassionately when patients are counterintuitively attached to self-destructive, ‘challenging’ ED behaviours.

5 Importantly, ED sufferers do not *want* to make themselves more ill. The term ‘ego-syntonic’ is another label that is often used in an unhelpful and reductionist way. The reality for ED sufferers is usually a lot more complicated and less black-or-white (i.e., it is not just ‘ego-dystonic’ versus ‘ego-syntonic’). There is a difference between ‘needing’ to perform ED behaviours (e.g., needing to starve oneself to relieve distressing emotions), and ‘wanting’ to perform ED behaviours (e.g., wanting to starve oneself to achieve desired emotions and fulfil values). Both inevitably intertwine and overlap, making it impossible to neatly separate them for the benefit of academic theories and treatment models.

## References

[1] Gaudiani JL, Bogetz A, Yager J (2022). Terminal anorexia nervosa: three cases and proposed clinical characteristics. J Eat Disord.

[2] Guarda AS, Hanson A, Mehler P, Westmoreland P (2022). Terminal anorexia is a dangerous term: it cannot, and should not, be defined. J Eat Disord.

